# Assessing horizontal equity in health care utilization in Iran: a decomposition analysis

**DOI:** 10.1186/s12889-020-09071-z

**Published:** 2020-06-12

**Authors:** Farideh Mostafavi, Bakhtiar Piroozi, Paola Mosquera, Reza Majdzadeh, Ghobad Moradi

**Affiliations:** 1 Social Determinants of Health Research Center, Kurdistan University of Medical Sciences, Sanandaj, Iran; 2 Department of Public Health and Clinical Medicine, Epidemiology and Global Health, Umeå University, Umeå, Sweden; 3 School of Public Health and Institute of Public Health Research, Epidemiology and Biostatistics, Tehran University of Medical Sciences, Tehran, Iran; 4 Department of Epidemiology and Biostatistics, Kurdistan University of Medical Sciences, Pasdaran Ave, Sanandaj, Iran

**Keywords:** Health inequalities, SOCIO-ECONOMIC, SOCIAL INEQUALITIES, HEALTH SERVICES

## Abstract

**Background:**

Despite the goal of horizontal equity in Iran, little is known about it. This study aimed i) to assess socioeconomic inequality and horizontal inequity in the healthcare utilization; and ii) to explore the contribution of need and non-need variables to the observed inequalities.

**Methods:**

This study used national cross sectional dataset from Utilization of Health Services survey in 2015. Concentration Index (C), Concentration Curve (CC) and Horizontal Inequity index (HI) were calculated to measure inequality in inpatient and outpatient health care utilization. Decomposition analysis was used to determine the contribution of need and non-need factors to the observed inequalities.

**Result:**

Results showed the pro-poor inpatient services in both rural (C = − 0.079) and non-rural areas (C = − 0.096) and the pro-rich outpatient services in both rural (C = 0.038) and non-rural (C = 0.007). After controlling for need factors, HI was positive and significant for outpatient services in rural (HI = 0.039) and non-rural (HI = 0.008), indicating that for given need, the better off especially in rural make greater use of outpatient services. The HI was pro-poor for inpatient services in both rural (HI = − 0.068) and non-rural (HI = -0.090), was significant only in non-rural area. Non-need factors were the most important contributors to explain inequalities in the decomposition analysis.

**Conclusion:**

Disentangle the different contribution of determinants, as well as greater HI in rural areas for outpatient and in non-rural areas for inpatient services, provide helpful information for decision makers to re-design policy and re-distribute resource allocation in order to reduce the socioeconomic gradient in health care utilization.

## Background

Equitable access and utilization of health services is one of the goals, tasks and challenges of governments [1]. Universal health coverage (UHC) is an important step toward achieving equity in the utilization of health services by all people [2, 3]. Typically, in high-income countries poorer individuals utilize more health care services due to need factors (i.e. lower health status). Conversely in low-income countries, poorer individuals are less likely to use services due to non-need factors (i.e. low income and lack of health insurance) and despite their greater need [4]. The principle of Universal health coverage (UHC) states that individuals with equal needs should utilize equal healthcare services [5, 6]. Therefore, as poorer individuals often face lower health status and greater need it is expected that they utilize more health services. but also to support the fullfilment of the UHC Monitoring horizontal equity is deemed necesary not only to provide a comprehensive picture of equity in health care [7, 8].

Iran, like many other countries, has set UHC and health equity as some of its main goals [9]. One of the first effort was the establishment of a public health care (PHC) network fully financed by the government in 1985 [10–12]. In 1989 The Social Security Act was enacted and the Social Security Organization was appointed as the institution responsible to implement and provide health care services for workers and persons covered by the Labor and Social Security Law. In addition, the Imam Khomeini Relief Foundation, was created by the government as a subsidy fund to cover inpatient services for poor and low-income individuals [10]. In 2005 the Family Physician Program and a Universal Health Insurance scheme were implemented with full financial support for rural areas, and partial financial support for urban residents [12, 13]. Lately, in 2014 the implementation of the Health Transformation Plan was an important step taken by the government (more focus on inpatient services in public sector) to achieve public health coverage through reducing the amount of out of pocket payment [14–16].

Little is known about equality in health care utilization in Iran, and equity was not studied nationally or methodologically. Previous studies in Iran have shown pro-rich inequalities in healthcare utilization, in which Sex, place of residence and health insurance coverage have been reported as the main predictors of observed inequalities [17–19]. On the same data of this study, a study using logistic regression models to analyze association between social variables with the self-reported need and usage of services in people who reported need; poor people reported more both of outpatient and inpatient needs than rich people, as well as usage of inpatient services was more in rich people and was not significant for outpatient services [20].

Equity in health care in Iran may therefore require being re-assessed and permanently monitored. To contribute and update the knowledge about horizontal equity in health care utilization in Iran, the present study aimed: i) to assess socioeconomic inequality and horizontal inequity in the utilization of health services; and ii) to explore the contribution of need and non-need variables to the observed inequalities.

## Methods

### Study design and participants

This study used national cross sectional dataset from Utilization of Health Services (UHS) survey in 2015. UHS was conducted by National Institute of Health Research under the supervision of the Statistical Centre of Iran and in coordination with the relevant departments in the Ministry of Health and Medical Education.

The target population of this study was a set of ordinary resident households (ordinary households are made up of several people who live together in a fixed residence, have the same expenditure and usually eat together) and group households, i.e. a group of people who all or most of them, due to their special circumstances, mainly have a common feature, have chosen a joint residence for their living and jointly manage the affairs of life in that residence. These were selected according to the latest general population and housing census of Iran in 2011. Institutional households such as student dormitories, barracks, and prisons were not included in the study.

The samples were selected using three-stage stratified probability sampling method; i) each province was classified into non-rural/rural geographic segment. ii) the non-rural areas were classified into two categories of “central city/non-central city” segment. iii) 20 households were selected from each segment using simple random sampling method. Which 10 households were selected as the main sample and 10 households were selected as the alternative sample.

The total number of segments in the whole country (m) is obtained by dividing the number of ordinary and group households by 10, and sample areas obtained from following formula:

1 $$ {m}_{th}=\frac{\sqrt{N_{th}}}{\sum \sqrt{N_{th}}}\times m\kern2.75em t=1,2,3,\dots, 31\kern0.5em ,h=1,2,3 $$

m_th_ is the number of sample areas in the h^th^ class of t^th^ province.N_th_ is the number of ordinary resident households living in the h^th^ class/category of the t^th^ provinces (from 31 provinces) based on the general census of population and housing in 2011.

A total of 22,470 households were enrolled in this study (*N* = 81,137 invited, *N* = 78,378 participated), and the response rate was 96.6%.

For the present study, resulting in a sample size of 12,944 individuals had been received health care from health care facility in the last 2 weeks, and 5404 individuals had been admitted to a hospital in the last year. Data were collected using a questionnaire via interviews. This study was conducted according to the guidelines laid down in the Declaration of Helsinki.

### Variable definition

Outcome variables were measured by health care utilization in inpatient and outpatient care, derived from the questions: “Have you been admitted to a hospital in the last year?” and “Have you received health care from a health care facility in the last two weeks?” respectively. Both variable coded as yes = 1 or no = 0.

To calculate the socioeconomic status variable, we used the data on a number of assets collected as part of the UHS survey. Using principal component analysis (PCA), an asset index was calculated for each of the subjects. We divided individuals based on “rank” instead of “weight” for the quintiles included in the decomposition. The index ranks people from the poorest to the richest, by classifying them into five quintiles: very poor, poor, moderate, rich, and very rich [21, 22].

Need factors included demographic variables (age and sex) and a health variable (self-reported health), used as proxies of need [4]. Age was categorized into three groups, less than 30 years, 30–59 and 60 years and older. Sex was defined as male/female. Self-reported health status variable was dichotomized into two groups good and poor. The information for this variable was derived from the question: “you had Have any major illness or suffered from any disability for at least the past year?” (Yes/No). Having either illness or disability was considered poor health and having no illness or disability was measured as good health status.

Non-need factors included socioeconomic status variables, education, basic and supplementary insurance, marital status, and occupation. Education was categorized into three groups: uneducated & elementary, middle & high school and college and above. Basic and supplementary insurance variables were both coded as yes = 1 or no = 0. Marital status was categorized into married or single and occupation or having job was defined as yes/no.

### Statistical analysis

First Concentration Index (C), Concentration Curve (CC) and Horizontal Inequity index (HI) were calculated to measure inequality in health care utilization. To form CC, individuals are sorted according to their socioeconomic status, then the cumulative percentage of population is plotted against the cumulative percentage of health variable. CC above (below) the line of equality indicate health variable is concentrated among poor (rich) individuals. C values range from + 1 to − 1. Positive (negative) value indicates the health variable is concentrated among rich (poor) individuals, and C equals zero means there is no inequality. To calculate the C, The Kakwani method was used by the following eq. (22): (Eq. 2)

2 $$ C=\frac{2}{\mu}\mathit{\operatorname{cov}}\left({y}_i,{R}_i\right) $$

Where C is concentration index, Cov is the Covariance, y_i_ is the health variable, R_i_ is the i^th^ individual’s fractional rank in the socioeconomic distribution and μ is the health variable mean. Wagstaff correction [23] to the C was used because of binary outcome variables.

Decomposition analysis was used to determine the contribution of need and non-need factors to the observed inequalitc we used the linear approximation of a probit model to estimate partial effects [22] (Eq. 3).

3 $$ {y}_i={\alpha}^m+{\sum}_j{\beta}_j^m{x}_{ji}+{\sum}_k{\gamma}_k^m{Z}_{k_i}+{\varepsilon}_i $$

The decomposition of the concentration index for *y*_*i*_, can thus be expressed as the following formula (Eq. 4):

4 $$ C=\sum \left(\frac{\beta_j^m{\overline{x}}_j}{\mu}\right){C}_j+\sum \left(\frac{\gamma_k^m{\overline{Z}}_k}{\mu}\right){C}_k+\frac{GC_{\varepsilon }}{\mu } $$

where, μ is the mean y_i_ (health care utilization), C_j_ and C_k_ are the concentration indices for X_j_ (need factors) and Z_k_ (non-need factors), ${\upbeta}_{\mathrm{j}}^{\mathrm{m}}$ and ${\upgamma}_{\mathrm{k}}^{\mathrm{m}}$ are the partial effects (d_y_/dx_j_, d_y_/dz_k_) for x and z, ${\overline{\mathrm{x}}}_{\mathrm{j}}$ and ${\overline{\mathrm{Z}}}_{\mathrm{k}}$ are the mean level of X_j_ and Zk, $\left(\frac{\upbeta_{\mathrm{j}}^{\mathrm{m}}{\overline{\mathrm{x}}}_{\mathrm{j}}}{\upmu}\right){\mathrm{C}}_{\mathrm{j}}$ and $\left(\frac{\upbeta_{\mathrm{j}}^{\mathrm{m}}{\overline{\mathrm{x}}}_{\mathrm{j}}}{\upmu}\right){\mathrm{C}}_{\mathrm{j}}$ are the contributions of need variables (j) and non-need variables (k), and $\frac{GC_{\varepsilon }}{\mu }$ is the generalized concentration index for the remaining error [22, 24].

Finally, the horizontal index was obtained from the concentration index presented by Eq. (1) minus the estimated contributions of the need variables calculated in Eq. (4). When the horizontal index (HI) is positive, the use of services by individuals with a higher socioeconomic status is more than their need, and when it is negative it indicates that the poor people of the community have received services more than their need [25]. The reference groups employed in the analysis were single, women, under the age of 30, who had college and above education, were in the highest socioeconomic quintile and had basic and complementary insurance. All analyses were performed on rural and non-rural separately.

## Results

**Fig. 1:**
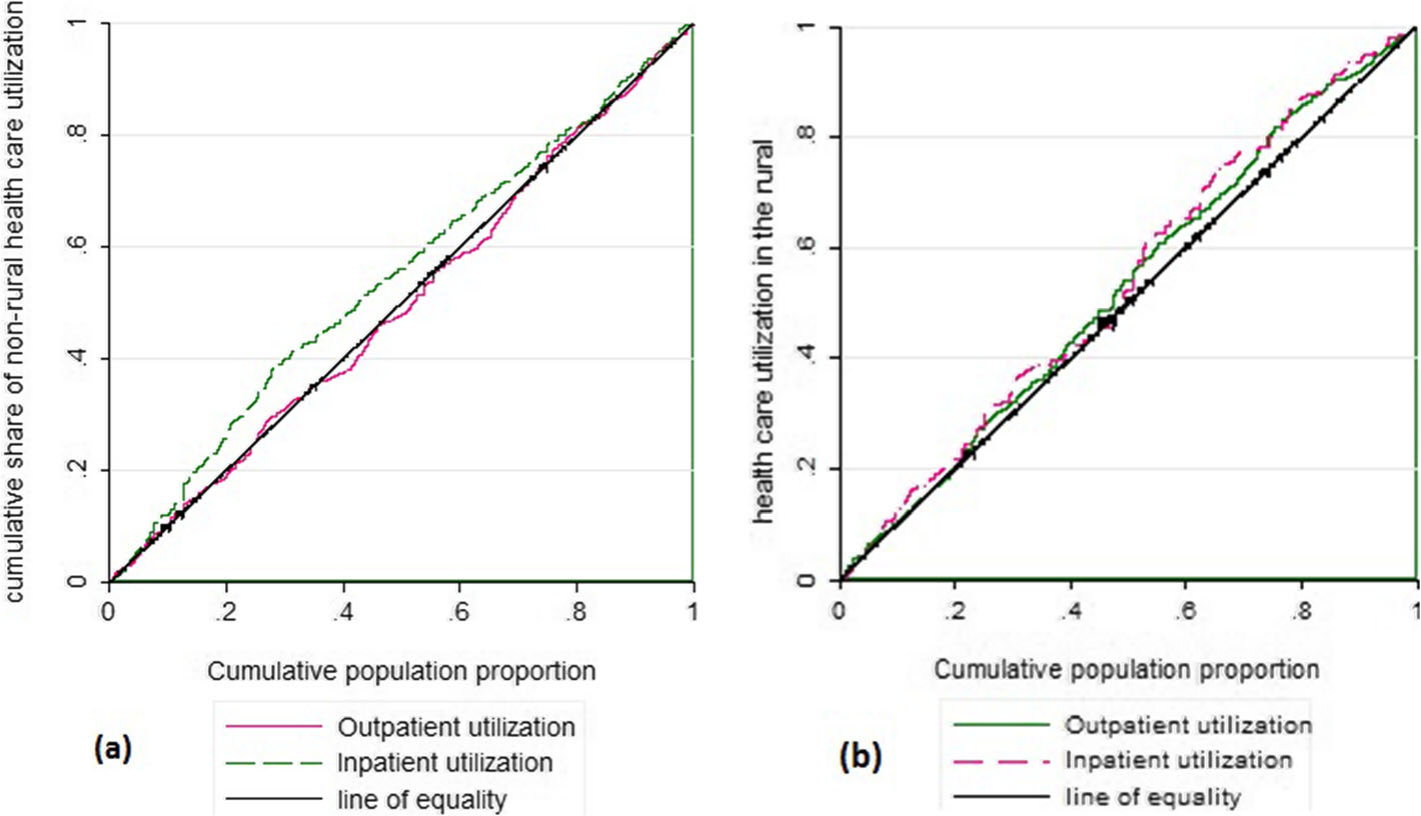
Concentration curve for outpatient and inpatient health care utilization in rural and non-rural

Table 1 shows the characteristics of the participants. The C for inpatient services were negative in both rural (C = -0.079) and non-rural areas (C = -0.096) pointing toward a higher utilization among individuals belonging to lower income households. On the other hand, the C for outpatient services were positive in both rural (C = 0.038) and non-rural (C = 0.007), which indicated concentration of these services in higher socioeconomic groups. The concentration curves in Fig. 1, confirmed what was indicated by the Concentration indexes.

**Table 1:** Variable characteristics

Characteristic	Non-rural (%)	Rural (%)
**Sex**		
Female	25,904(49.32)	12,753(49.32)
Male	26,616(50.68)	13,105(50.68)
**Education**		
Uneducated & Elementary	25,456(52.61)	17,985(77.20)
Middle & High school	12,830(26.52)	3877(16.64)
College and above	10,099(20.87)	1435(6.16)
**Socioeconomic Status (SES)**		
Poorest SES	10,818(21.06)	4773(19.59)
2th SES	9587(18.66)	4646(19.07)
Middle SES	9960(19.39)	4492(18.43)
4th SES	10,446(20.33)	4308(17.68)
5th SES	10,566(20.57)	6150(25.24)
**Basic insurance**		
Yes	46,580(88.69)	24,469(94.63)
No	5940(11.31)	1389(5.37)
**Supplementary Insurance**		
Yes	11,617(24.58)	1736(7.07)
No	35,645(75.42)	22,828(92.93)
**Age**		
< 30	26,878(51.18)	14,215(54.97)
30–59	20,292(38.64)	8788(33.99)
≥ 60	5350(10.19)	2855(11.04)
**Marital status**		
Single	18,455(40.54)	8601(39.85)
Married	27,068(59.46)	12,981(60.15)
**being employed**		
Yes	27,163(65.60)	12,188(63.13)
No	14,241(34.40)	7118(36.87)
**Health status**		
Good	46,690(88.90)	22,875(88.46)
Poor	5830(11.10)	2983(11.54)
**Outpatient utilization**		
Yes	8743(16.65)	4441(17.17)
No	43,777(83.35)	21,417(82.83)
**Inpatient utilization**		
Yes	3582(6.82)	1181(7.00)
No	48,938(93.18)	24,047(93.00)

After controlling for need factors, the HI was positive and significant for outpatient services in rural (HI = 0.039) and non-rural areas (HI = 0.008), indicating that for given need, the better off make greater use of outpatient services. The HI remained negative for inpatient services in rural (HI = -0.068) and non-rural areas (HI = -0.090), which was significant only in non-rural area indicating that the inpatient services were more utilized by the poor groups (Table 2).

**Table 2:** Decomposition of Concentration Index for Inpatient health care utilization in rural and non-rural

	Rural					Non-rural				
	Coef	Elast	CI	Cont to C	C%	Coef	Elast	CI	Cont to C	C%
Age										
< 30										
30–59	0.006^*^	0.030	−0.032	−0.0009	1.23	−0.000	− 0.004	− 0.039	0.0001	− 0.17
≥ 60	0.014^***^	0.022	0.012	0.0002	−0.34	0.005	0.007	0.016	0.0001	−0.13
Sex										
Female										
Male	0.000	0.004	−0.007	−0.000	0.047	−0.003^*^	− 0.029	− 0.008	0.0002	− 0.26
Health statue										
Good										
Poor	0.71^***^	1.180	−0.008	− 0.010	12.90	0.733^***^	1.193	−0.005	−0.006	6.40
Subtotal need				−0.010	13.83				−0.005	5.84
Education										
College and above										
Middle & High school	0.000	0.000	−0.028	−0.000	0.003	0.809	−0.002	− 0.025	0.000	− 0.05
Uneducated & Elementary	0.000	0.003	0.203	0.000	−0.009	−0.003	− 0.026	0.188	− 0.005	5.22
Basic insurance										
Yes										
No	−0.007	− 0.005	0.007	−0.000	0.05	−0.015	− 0.025	0.029	− 0.000	0.76
Supplementary insurance										
Yes										
No	−0.007	− 0.095	0.333	−0.031	40.10	−0.005	− 0.064	0.258	− 0.016	17.22
Being employed										
Yes										
No	−0.000	−0.005	− 0.012	0.000	− 0.07	0.005^**^	0.029	−0.011	− 0.000	0.36
Marital status										
Single										
Married	−0.001	− 0.009	0.011	−0.000	0.13	0.005^**^	0.046	0.024	0.001	−1.16
Economic statue										
Poorest SES	0.013^**^	0.044	−0.227	− 0.010	12.69	0.003	0.032	−0.540	−0.017	18.03
2th SES	0.170^***^	0.050	−0.083	−0.004	5.38	−0.000	− 0.000	0.057	− 0.000	0.00
Middle SES	0.023^***^	0.071	0.024	0.001	−2.16	0.004	0.006	0.068	0.000	−0.48
4th SES	−0.002	− 0.007	0.098	− 0.000	0.91	0.000	0.000	0.765	0.000	−0.07
5th SES										
Subtotal non-need				−0.044	57.024				− 0.038	39.83
Total				−0.099					−0.043	
Residual				0.020					−0.053	
C				−0.079^***^					−0.096^***^	
HI				−0.068					−0.090^***^	

Coeff Marginal effects, Elast elasticity, CI Concentration index of the social determinants, Cont to C Contribution to the overall concentration index, C% unadjusted percentage calculated on the overall explained portion of the C, HI Horizontal Index

* 0.01 ≤ *p* < 0.05; ** 0.001 ≤ *p* < 0.01; *** *p* < 0.001

Table 2 (inpatient services) and Table 3 (outpatient services) show the results of the decomposition analysis by non-rural and rural areas. The first column, regression coefficients show the partial effect of each variable on the utilization. The second column indicates the elasticity of health care utilization for each determinant. The third column shows the concentration index of each of the determinants included in the analysis. The two last columns show the absolute and percentage contributions of each factors to the overall concentration index.

**Table 3:** Decomposition of Concentration Index for Outpatient health care utilization in rural and non-rural

	Rural					Non-rural				
	Coef	Elast	CI	Cont to C	C%	Coef	Elast	CI	Cont to C	C%
Age										
< 30										
30–59	−0.014^***^	− 0.029	− 0.032	0.000	2.45	−0.000	− 0.001	− 0.039	0.000	0.63
≥ 60	−0.015^***^	− 0.009	0.012	−0.000	− 0.30	− 0.004	− 0.003	0.016	− 0.000	− 0.70
Sex										
Female										
Male	−0.007^*^	−0.022	−0.007	0.000	0.45	0.001	0.003	−0.008	−0.000	− 0.46
Health statue										
Good										
Poor	0.320^***^	0.215	−0.008	−0.001	−4.86	0.328^***^	0.219	−0.005	−0.001	−15.77
Subtotal need				−0.000	−2.26				−0.001	−16.30
Education										
College and above										
Middle & High school	−0.006	− 0.006	− 0.028	0.000	0.45	0.002	0.004	−0.025	−0.000	−1.45
Uneducated & Elementary	0.007	0.032	0.203	0.006	17.05	0.004	0.013	0.188	0.002	35.15
Basic insurance										
Yes										
No	−0.039^***^	−0.012	0.007	−0.000	−0.25	0.000	0.000	0.029	0.000	0.25
Supplementary insurance										
Yes										
No	0.015^**^	0.085	0.333	0.028	74.05	0.009^***^	0.040	0.258	0.010	147.63
Being employed										
Yes										
No	0.016^***^	0.035	−0.012	− 0.000	−1.14	− 0.003	− 0.007	−0.011	0.000	1.28
Martial statue										
Single										
Married	−0.005^**^	−0.019	0.011	−0.000	− 0.60	− 0.008^**^	− 0.029	0.024	− 0.000	−9.89
Economic statue										
Poorest SES	0.001	0.001	−0.227	− 0.000	− 0.96	0.005	0.019	− 0.540	− 0.010	− 149.42
2th SES	− 0.001	− 0.002	− 0.083	0.000	0.51	0.009	0.009	0.057	0.000	7.93
Middle SES	−0.013^**^	−0.016	0.024	−0.000	−1.02	0.004	0.002	0.068	0.000	2.61
4th SES	0.018^***^	0.018	0.098	0.001	4.87	0.021^***^	0.012	0.765	0.000	12.90
5th SES										
Subtotal non-need				0.035	92.96				0.003	46.99
Total				0.0358					0.002	
Residual				0.002					0.005	
C				0.038^*^					0.007	
HI				0.039^***^					0.008^***^	

Coeff Marginal effects, Elast elasticity, CI Concentration index of the social determinants, Cont to C Contribution to the overall concentration index, C% unadjusted percentage calculated on the overall explained portion of the C, HI Horizontal Index

* 0.01 ≤ *p* < 0.05; ** 0.001 ≤ *p* < 0.01; *** *p* < 0.001

Regarding the utilization of inpatient care, there was a significant positive association between utilization of services and older age, poor health and low SES in rural residents. In non-rural area, being married, unemployed and individuals who reported poor health, were more likely to use services. Male, people in middle age (30–59) and poor health status were all concentrated among the poor individuals. Need factors explained a smaller proportion of the inequality favoring the poor in both rural and non-rural areas (13.83 and 5.84% respectively), while non-need factor accounted for bigger proportion of the inequality (57.024 and 39.83% in rural and non-rural areas, respectively). Among the need factors, poor health was the major contributor, whereas the other factors displayed an insubstantial role. Among the non-need factors, lack of supplementary insurance and low SES made the largest contributions to explain the pro-poor inequalities in both rural and non-rural areas.

For the utilization of outpatient care, in rural areas, there was a negative association between older age, sex, marital status and lack of basic insurance and a positive association between high SES, poor health, being unemployed and lack supplementary insurance with use of this service. On the other hand, in non-rural areas, individuals with poor health, high SES and lacking supplementary insurance were more likely to use the services. The need factors were slightly offsetting (− 2.26% and − 16.30% in rural and non-rural, respectively) the contribution of non-need factors which in this case accounted for most of the inequality favoring the better-off (92.96 and 46.99% in rural and non-rural, respectively). The lack of supplementary insurance coverage was the largest non-need contributor in both rural and non-rural area, with an additional contribution coming from education. Interestingly, high SES made a very small contribution to the pro-rich inequalities in rural area while low SES was instead offsetting the inequalities in non-rural area.

## Discussion

The results of this study suggest firstly, that whereas inpatient services are fairly equitable and seem to meet the principle of horizontal equity, the use of outpatient services is substantially concentrated among the well-off population. Second, that rural areas displayed lower levels of inequality in the use of inpatient services while non-rural areas showed lower levels of inequalities in the use of outpatient services. Third, the decomposition analysis suggested that non-need factors were the most important contributors to explain both inpatient and outpatient inequalities, and among them, the lack of supplementary insurance and SES were the most important explanatory factors.

The overall observed pattern of inequality in outpatient and inpatient healthcare services in our study is in accordance with the findings of studies conducted in three high-income countries in East Asia (Honk Kong, South Korea, and Taiwan) [26] and Brazil [27]. On the one hand, private insurance coverage (Hong Kong and Brazil), low education, unemployment (South Korea), place of residency and income (Taiwan) were the main explanatory factors for outpatient pro-rich inequalities. On the other hand, policy interventions as for example services-on-wheel and exemption of co-payment in rural residents were driving the pro-poor inpatients inequalities [26, 27]. Contrasting patterns to those found in the present study have also been described in other settings. For example a study in China reported pro-rich inequity of inpatient utilization in rural residents [28]. Our study adds to this meagre literature by suggesting the levels of inequalities in the use of inpatient services among rural residents is lower than in the non-rural ones. Possible explanations of our findings could be the successful implementation of the family physician program, the rural insurance scheme and the referral system, which have already shown increased equal and comfortable access to health services [11, 29]. As a result of these interventions, family physicians act as a gatekeeper to the system and rural insurance holders only pay a small portion of the total costs when they are admitted to hospitals through the referral system. Conversely, in large cities, family physicians doesn’t have an obvious role as gate-keepers [30].

This study also suggested that utilization of outpatient service is not equitable among Iranian population. In our study, the pro-rich inequality in outpatient services was higher in rural than in non-rural areas. This inequality was mainly explained by the higher level of utilization among people with no supplementary health insurance, which was in fact concentrated among the high SES. In Iran, there are three type of health care services including; public sectors, quasi-public sectors and private sectors. While hospitalization services are mainly provided by the public sector (more than 70% of inpatient facilities and services), more than 70% of outpatient facilities and services are provided by the private sector [31]. Private sector fee is much higher than the public sector’s, which therefore could lead to pro-rich outpatient services. Despite increasing the share of government and insurance funds in total health expenditures, finding has shown the out of pocket payments of the households increased even more than the previous years [15]. After the implementation of the Health transformation Plan, private sector services fee increased by an average of over 100% [32]. In addition, part of the outpatient service including dental services and rehabilitation services are not covered by insurance and have also been reported to be pro-rich [31, 33]. Despite the high insurance coverage in Iran, service coverage and cost coverage by insurances are not sufficient when an individual need to use the private sector, which consequently has led to reduced access and utilization of services among the low-income individuals [34]. Also evidence of other studies in Iran have shown that low quality of health care delivery by public sectors and family physicians led to seeking care in private sectors and specialist services particularly in outpatient care [35, 36]. Similar to our findings, a previous study in Iran showed that poor socioeconomic status was associated with low utilization of services [37]. In contrast of our study and other studies in Iran, a local study conducted in 2012 in Shiraz (the fifth most populous city of Iran) reported a pro-poor inequality in utilization of outpatient services after standardizing for need factors. The allocation of subsidies and low cost of services in the public sector, high insurance coverage, and financial barriers related to upper level access, reported as the main factors associated to the pro-poor inequality; in addition the reason reported for under-utilization of the rich individuals is low quality of services in Shiraz [38].

This study has some limitations; the data on socioeconomic status, health status, and the utilization of services were collected via self-reported questionnaire, as a result, the collected data might have some bias. In the present study a PCA analysis was used to calculate socioeconomic variable therefore the choice of variables and the appropriateness of the weights assigned to them might be amatter of concern [4]. In addition, the self-reported health variable was binary (good and poor health), which will not indicate variation in health status and might not adequately discriminate those in need for health care services, therefore its effect may be overestimated or underestimated. Another limitation is big residuals in some of the models meaning the variables included in the model were not able to adequately explain the inequalities in the outcome. Some other variables i.e. quality of health care delivery could have been relevant to explain these inequalities, however information was not available in the dataset. Also to handle the limitation of secondary data, survey method including the survey instrument was considered. Concerning the analysis, we used the correction proposed by Wagstaff et al. to the concentration index because of the binary nature of the health outcome [4].

## Conclusions

Our results, suggest a pro-poor income-related inequality in inpatient and pro-rich income-related inequality in outpatient services. Inequalities are mainly explained by non-need factors i.e. lack of supplementary insurance and SES. Magnitude of HI was greater in rural areas for outpatient services and greater in non-rural area for inpatient services. Disentangle the different contribution of determinants as well as variations in HI among rural and non-rural areas provide helpful information for decision makers to re-design policy and re-distribute resource allocation in order to reduce the socioeconomic gradient in health care utilization.

## Data Availability

The datasets used and/or analyzed during the current study are available from the corresponding author on reasonable request.

## Abbreviations

C: Concentration Index
CC: Concentration Curve
HI: Horizontal Inequity index
UHC: Universal health coverage
PHC: Public health care
UHS: Utilization of Health Services
PCA: Principal component analysis
